# Study on the Microstructure and Properties of Welded Joints of Laser Shock Peening on HC420LA Low-Alloy High-Tensile Steel

**DOI:** 10.3390/ma16124238

**Published:** 2023-06-08

**Authors:** Yu Wang, Aixin Feng, Xiaoming Pan, Chunlun Chen, Yacheng Wei, Jun Wang

**Affiliations:** 1 College of Mechanical and Electrical Engineering, Wenzhou University, Wenzhou 325035, China; 20461439016@stu.wzu.edu.cn (Y.W.); 00132004@wzu.edu.cn (X.P.); 20451438045@wzu.edu.cn (C.C.); 20451438039@wzu.edu.cn (Y.W.); 2 Rui’an Graduate College, Wenzhou University, Wenzhou 325200, China; 3 Zhejiang Y-Hu Auto Parts Co., Ltd., Wenzhou 325035, China; 20461439072@stu.wzu.edu.cn

**Keywords:** laser shock peening, low-alloy high-strength steel, microstructure, mechanical properties

## Abstract

Laser shock peening is a promising surface strengthening technology that can effectively improve the mechanical properties of materials. This paper is based on the laser shock peening process for HC420LA low-alloy high-strength steel weldments. Contrast analysis of the evolution of the microstructure, residual stress distribution and mechanical properties of the welded joints before and after the laser shock peening on each region is carried out; a combination of tensile fracture and impact toughness fracture morphology analyses of laser shock peening on the welded joint strength and toughness regulation mechanism are also completed. The results show that the laser shock peening can refine the microstructure of the welded joint effectively, the microhardness of all areas of the joint increases and the weld residual tensile stresses are transformed into beneficial residual compressive stresses, affecting a layer depth of 600 μm. In addition, the strength and impact toughness of welded joints of HC420LA low-alloy high-strength steel are improved.

## 1. Introduction

Low-alloy high-strength steel has become a key material for automotive light weighting such as seat skeletons because of its high specific strength, good processing properties and high ductility [1,2,3]. Seat frames are often welded from thin-walled parts, complex structures and welding position; the mechanical properties of the welded joint are affected by the characteristics of its welding process. In the “melt before connecting”, and due to welding heat cycle effects, low-alloy high-strength steel welded joints experience residual tensile stresses, uneven microstructure, heat-affected zone softening and other defects [4]. This causes the joint’s resistance to deform and toughness to decline; hence, the joint will have the potential for fracture damage by dynamic load during driving. Welded joints are the weak point of the seat skeleton; therefore, improving the mechanical properties of the joint has attracted increasing attention [5].

In order to decrease the defects of welding and increase their service life, various post-weld strengthening processes have been developed. They generally use heat treatments, chemical treatments, mechanical laminating and other methods to improve the mechanical properties of the joint [6,7]. Each has its own advantages, but they have a series of problems, such as high cost, low production efficiency and poor performance stability, which cannot meet the needs of modern technology and social manufacturing. Laser shock peening is a surface strengthening technology that has emerged in recent years. Compared with traditional surface strengthening technology, it has the significant advantages of non-contact, precise and controllable strengthening area, low cold work hardening and deep stress field, thus emerging as the current surface strengthening technology [8,9,10,11] by refining the microstructure of the surface layer of the material and inducing residual compressive stress to improve the problems of uneven organization and poor mechanical properties [12,13,14,15].

Laser shock peening has been widely used in the surface modification of materials, and many scholars have tried to study the regulation of laser shock peening on welded joints [16,17,18,19]. The microstructure and residual stresses of welded joints can be significantly enhanced by changing the process parameters of laser shock peening, thus improving the mechanical properties of the joints. Therefore, the aim of this paper is to investigate the effects of laser shock peening on the tissue–stress complex strengthening mechanism of HC420LA high-strength steel welded joints and the regulation of joint strength and toughness.

## 2. Materials and Methods

### 2.1. Material and Welding Process

The test used HC420LA low-alloy high-strength steel provided by Shanghai Baosteel Company. The chemical composition is shown in Table 1. The size of the welded specimen was 150 mm × 75 mm × 1.5 mm. The laser welding equipment is provided by Shenzhen Jushun Laser Technology Co., Ltd. (Shenzhen, China). The laser welding process parameters are as follows: laser power 1.8 Kw, welding speed 2.5 m/min and defocusing distance +1.5 mm. Argon gas was used as shielding gas.

### 2.2. LSP Process

**Figure 1:**
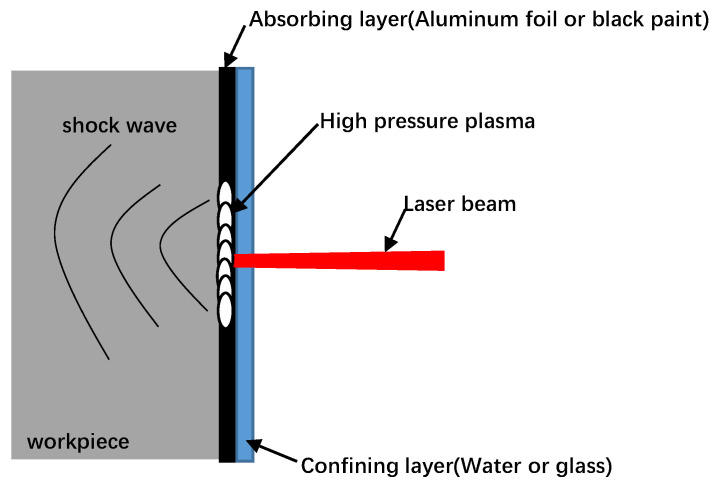
Schematic diagram illustrating the laser shock peening process.

**Figure 2:**
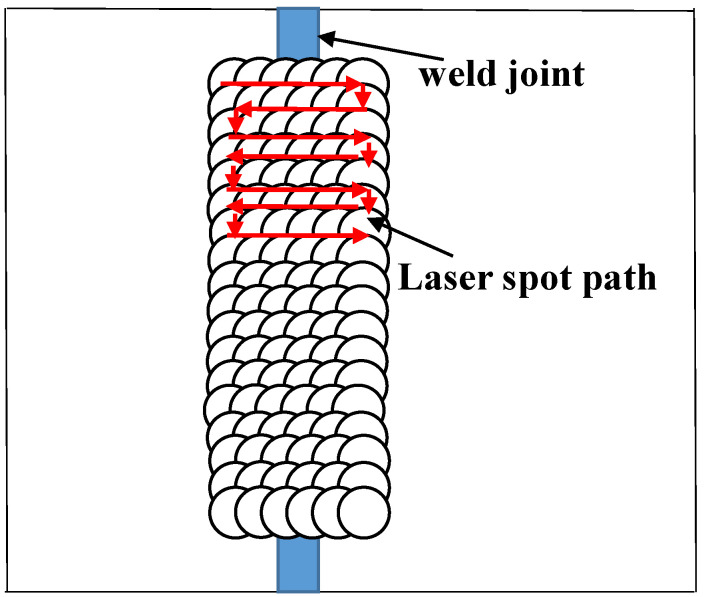
Processing path of laser shock peening.

Before laser shock peening, the welded parts were sanded and polished using different levels of Si sandpaper, placed in an ultrasonic cleaner containing alcohol and then air-dried. The test was conducted using laser shock peening equipment provided by the Shenyang Institute of Automation. The LSP main parameters are the following: laser power density 8.49 GW/cm^2^, 15 ns pulse width, 3 mm diameter circular spot, lap rate of 50%, deionized water as the confining layer and black paint as the absorbing layer. The principle of laser shock peening is shown in Figure 1, where the laser shock peening processing path is shown in Figure 2.

### 2.3. Characterization

**Figure 3:**
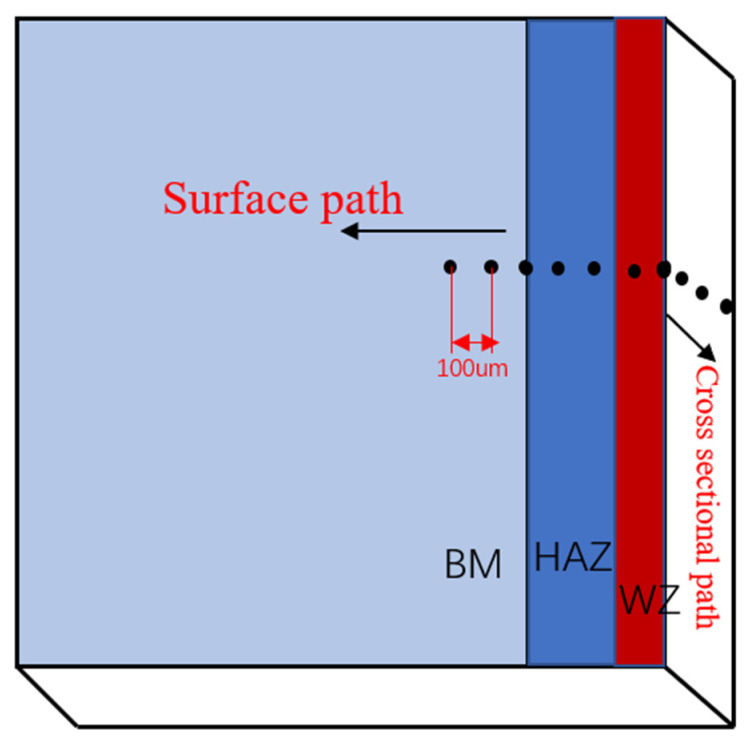
Path of microhardness and residual stress measurement.

Microstructure analysis was completed using an optical microscope (BX53M OM) from Tokyo, Japan, residual stress analysis with X-ray diffraction (LXRD) from PROTO (Waterloo, ON, Canada), microhardness (HXD-1000TM/LCD) from Shanghai, China, tensile tests (INSTRON 3369) from Norwood, MA, USA, an impact toughness test (PTM2200-D1) from Shenzhen, China; a scanning electron microscope (TESCAN Mira 3 XH) from TESCAN Brno, Brno, Czech Republic was used to observe the fracture morphology. The paths of residual stress and microhardness before and after LSP measurement are shown in the Figure 3. The depth direction hardness and residual stress are measured after electrolytic polishing. The Charpy impact test used a model PTM2200-D1 pendulum impact tester. The test was conducted at room temperature with a nominal energy of 300 J, voltage of 380 V, power of 1 kW and pendulum lifting angle of 150° ± 1°. Since the thickness of the specimen was only 1.5 mm, it did not meet the thickness requirement of the national standard GB/T 19748-2005 “Charpy V-notch pendulum impact test apparatus for steel”. A 4 mm thick pad was added on both sides of the specimen during the test. After the 2 mm deep v-notch impact in the heat affected zone, the impact power value was recorded on the display of the equipment.

## 3. Results and Discussion

### 3.1. Microstructure

**Figure 4:**
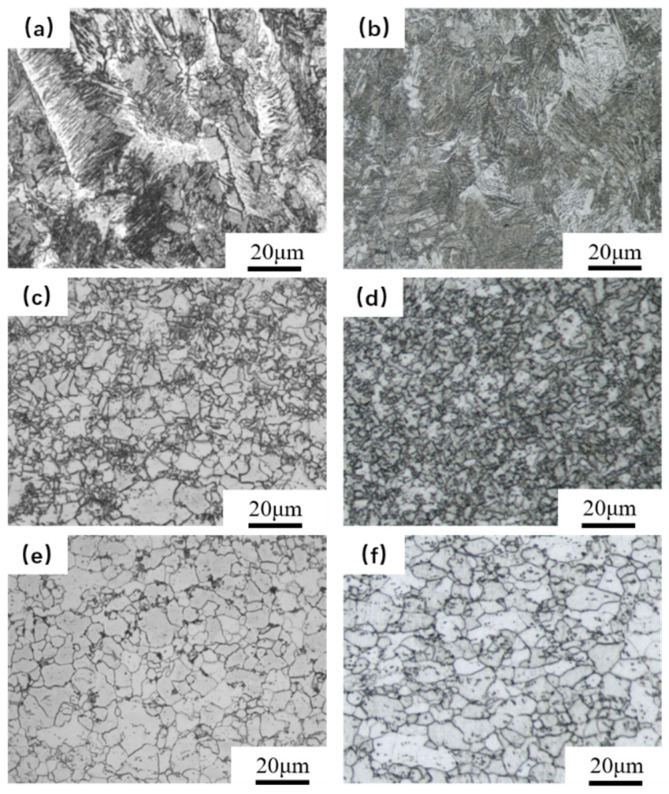
Microstructure of the HC420LA laser-welded joint before and after LSP. (**a**) Weld zone of untreated specimens; (**b**) Weld zone after LSP; (**c**) Heat-affected zone of untreated specimens; (**d**) Heat-affected zone after LSP; (**e**) Base material of untreated specimens; (**f**) Base material after LSP.

Figure 4 shows the HC420LA low-alloy high-strength steel laser welding head microstructure distribution before and after laser shock peening. Figure 4a shows the weld seam microstructure of untreated specimens; due to the thermal cycling effect of laser welding, the region is mainly a mixture of ferrite and bainite microstructures, with side lath ferrite along the columnar crystal inward growth and irregularly shaped grains of bainite in the ferrite around the distribution. Figure 4b shows the weld after laser shock peening. Compared with the untreated specimen, the needle-like ferrite is distributed in a network and has no obvious directionality. This is due to the formation of a piece of ferrite in the side slat ferrite caused by plastic deformation as a result of the laser’s impact. The heat-affected zone microstructure characteristics of the untreated specimen show a small area of fine grains uniting the distribution of large grains, resulting in a reduction in the strength and toughness of the region due to the thermal cycling of laser welding and the impact of rapid cooling; this is shown in Figure 4c. Figure 4d shows the microstructure of the heat-affected zone of the joint after laser shock peening. The grain size refinement resulted in more dislocations and dislocation entanglement. This phenomenon can be explained by the mechanism of dislocation movement: when the plastic deformation generated by laser shock peening reaches a certain level, dislocation occurs, dividing the grains into smaller sizes or the so-called fine grain reinforcement, which also allows the hardness of the welded heat-affected zone to increase. As shown in Figure 4e, the microstructure of the base material area for the untreated specimens is mainly ferrite, with a small amount of pearlite and precipitates; the grain size distribution is not uniform and their attachment to the ferrite grain boundaries of the carbide cause an increase in hardness. Figure 4f shows the microstructure of the base material area of the joint after laser shock peening. The grain distribution is more intensive than the former. The grain size does not change much compared to the former, while the carbide is more uniformly dispersed, and hardness is improved. Since laser shock peening is a cold process, the microstructure of the welded joint does not undergo a phase change. According to Hall–Petch theory, mechanical properties such as the tensile strength of the material will be improved with the refinement of grains within a certain range, while the increase in ferrite refinement also improves the impact toughness of the weld zone.

### 3.2. Residual Stresses

**Figure 5:**
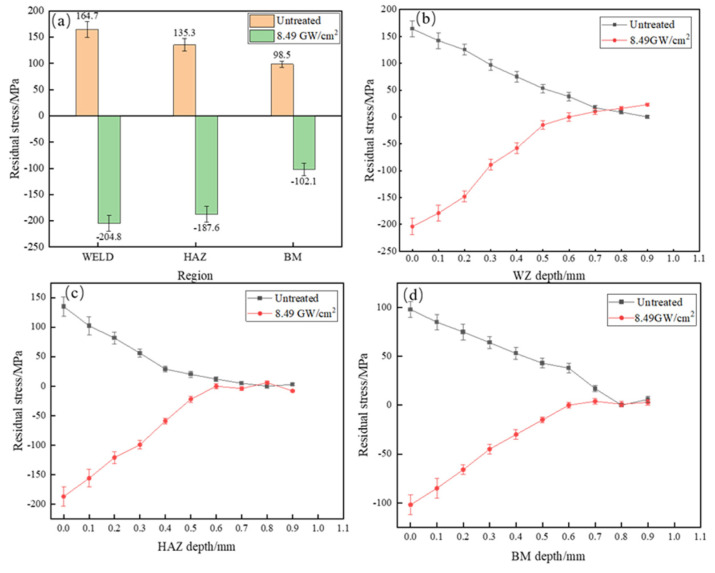
Residual stress distribution of the HC420LA laser−welded joint before and after LSP. (**a**) Welded joint surface residual stress distribution; (**b**) Weld zone depth direction residual stress distribution; (**c**) Heat–affected zone depth direction residual stress distribution; (**d**) Base material zone depth direction residual stress distribution.

Figure 5 shows the distribution of residual stresses in laser-welded joints of HC420LA low-alloy high-strength steel before and after laser shock peening. Figure 5a shows the residual stress distribution on the surface of each region of the joint after laser welding of HC420LA high-strength steel joints in each region of the surface residual stress performance for residual tensile stress. The welding process on the joint of the thermal cycle of each zone is not uniform which results in differences in the organization of each zone; this is also due to the thermal expansion and contraction of the joint metal by the rigid constraint of the joint and residual tensile stresses. The weld residual tensile stresses in the weld zone, heat affected zone and base material zone of the joint are 164.7 MPa, 135.3 MPa and 98.5 MPa. The residual stress values in each region of the joint after laser shock peening are −204.8 MPa, −187.6 MPa and −102.1 MPa, which are 2.24 times, 2.39 times and 2.04 times higher when compared to the untreated joint. As shown in Figure 5b for laser shock peening on the HC420LA low-alloy high-strength steel laser-welded joints in the depth direction of the change, the residual compressive stress generated by the laser shock peening on the surface of the welded joint reaches a maximum value, and the induced residual compressive stress gradually decreases along the depth direction and eventually tends towards residual tensile stress; this is consistent with the findings of the literature [20,21]. The depth of the laser-induced residual stress field in laser-welded joints of HC420LA low-alloy high-strength steel by LSP reaches about 600 μm. Figure 5c,d show the depth residual stress distribution in the heat-affected zone and the base material zone, respectively. The increase in residual stress in the weld zone and the base material zone is also due to the high-pressure shock wave generated by laser shock peening that breaks the original bainite tissue with coarse ferrite, resulting in plastic deformation of the material, making the size and grain size of the tissue reduced and refined.

### 3.3. Microhardness

**Figure 6:**
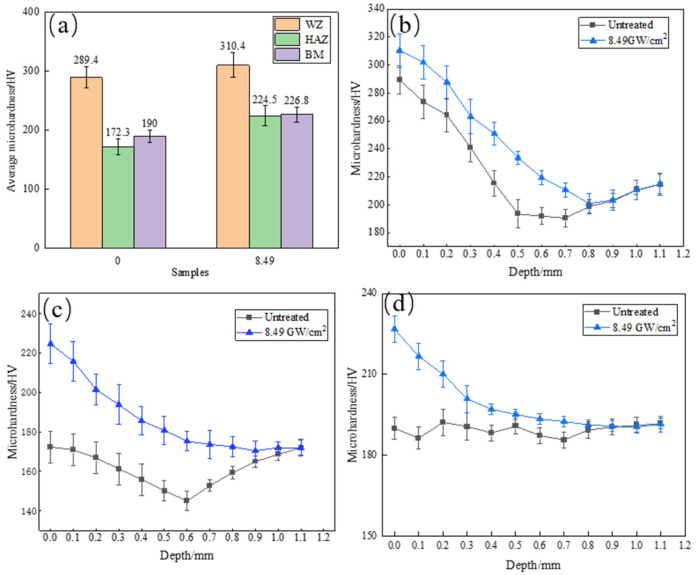
Microhardness distribution of the HC420LA laser-welded joint before and after LSP. (**a**) Welded joint surface microhardness distribution; (**b**) Weld zone depth direction microhardness distribution; (**c**) Heat–affected zone depth direction microhardness distribution; (**d**) Base material zone depth direction microhardness distribution.

Figure 6 shows the hardness distribution of the surface and depth of each region before and after laser shock peening on HC420LA high-strength steel welded joints. Figure 6a shows that the laser shock peening on HC420LA high−strength steel laser-welded joints after the microhardness of each region significantly increases. The trend of hardness distribution is still the weld zone along the direction of the parent material zone, which gradually decreases. The weld zone has the highest hardness value, followed by the untreated joints’ weld zone, heat-affected zone and the parent material zone with an average surface microhardness of 289.4 HV, 172.3 HV and 190. With a laser impact power density of 8.49 GW/cm^2^, the joint areas are 310.4 HV, 224.5 HV and 226.8 HV; compared to untreated specimens, there was an increase of 7.3%, 30.3%, 19.4%, with the joint heat-affected zone ground microhardness experiencing the most significant increase. This is due to the HC420LA high−strength steel laser welding head heat–affected zone coarse grains in the laser shock wave induced by effective refinement. Figure 6b,c show the laser-welded and heat-affected zone depth direction of the microhardness distribution of HC420LA low-alloy high−strength steel after LSP. The depth direction of the joint microhardness at the surface hits a maximum and gradually decreases along the depth direction; then, it increases into a “V”−shaped distribution, which is due to the fact that the upper and lower ends of the joint are cooled faster, and the grains are finer, so the hardness at both ends of the joint is higher than that at the middle part. Figure 6d shows the hardness distribution in the depth direction of the base material area; the inverse bremsstrahlung effect caused by the laser shock peening makes the change insignificant.

### 3.4. Tensile Fracture Morphology

**Figure 7:**
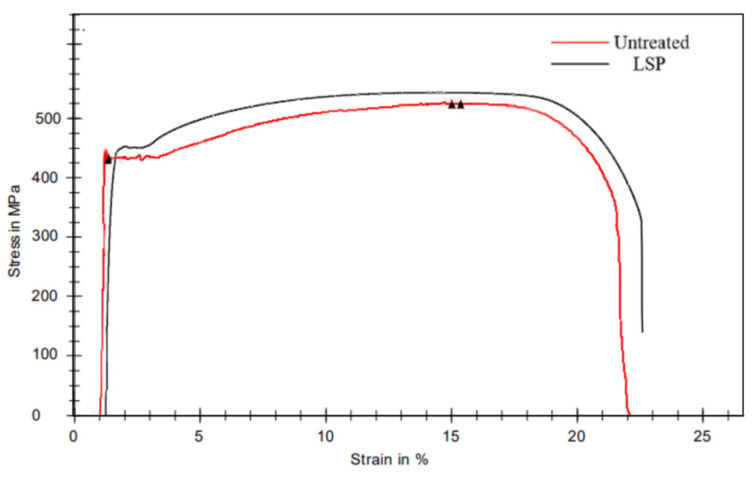
The tensile stress curves of the specimens before and after LSP.

Figure 7 shows the tensile stress curves of the specimens before and after laser shock peening. Untreated HC420LA low-alloy high−strength steel laser-welded joints have a tensile strength of 523 MPa; after the laser power density of 8.49 GW/cm^2^ treatment, the tensile strength is 547.4 MPa. Compared to the untreated joints, the tensile strength increased by 4.7%. The grain refinement and grain boundaries occurring in the surface layer of the HC420LA low-alloy high-strength steel welded joints effectively prevent the sliding of new dislocations during the tensile process [22,23].

**Figure 8:**
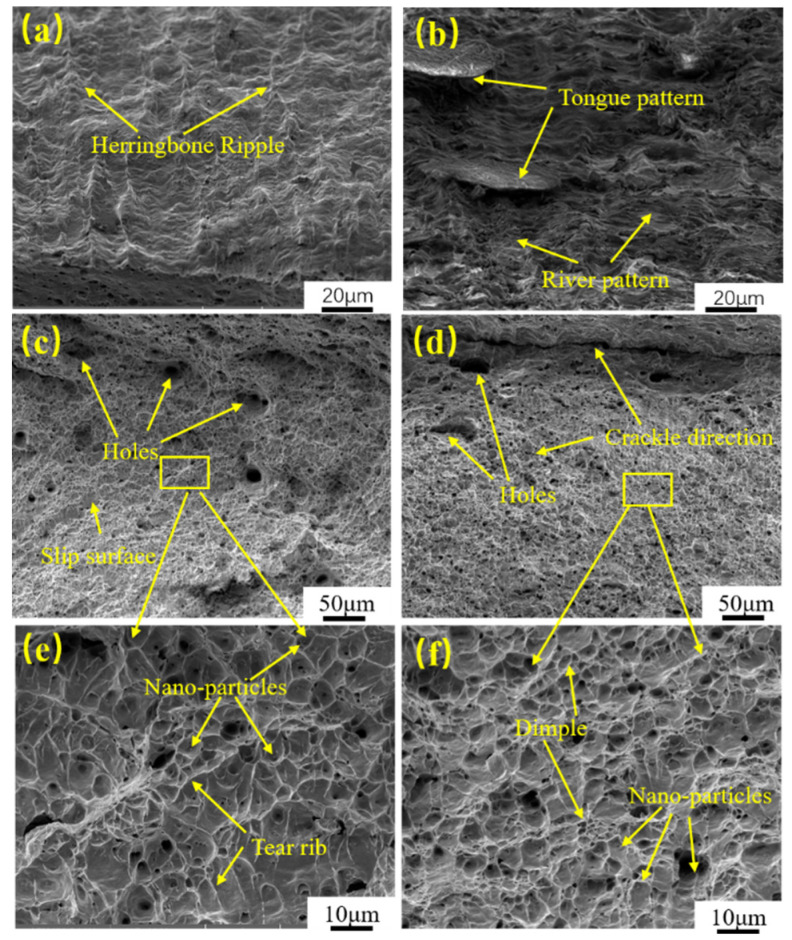
Tensile fracture morphology of HC420LA-welded joints before and after LSP; (**a**) Shear zone of untreated specimens; (**b**) Shear zone after LSP; (**c**) Fiber zone of untreated specimens; (**d**) Fiber zone after LSP; (**e**) Local magnification of the fiber area of the untreated specimen; (**f**) Local magnification of the fiber area after LSP.

Figure 8 shows the tensile fracture morphology of HC420LA high-strength steel laser-welded joints before and after laser shock peening, The shear zone shown in Figure 8a,b is mainly a ripple-like slip zone. Figure 8c,d shows that the tensile fracture morphology is microporous with tough nests; it lies between the characteristics of a typical tough nest micro-poly-porous fracture. Figure 8e is an enlarged view of the fiber zone. Figure 8c shows the fracture; the size of the tough nests is generally 1–2 μm, with a few larger holes scattered locally. The size and depth of the tough nests are more uniform. Some large size tough nests are wrapped with small size tough nests. The macroscopic fracture in Figure 8d has a “step” fracture, which may be due to the presence of stress concentration at this point, coupled with the cracks sprouting and then converging into a step shape. Some of the tough fractures are upwardly oriented and extremely shallow at this point, surrounded by dense tearing ribs and micropores. This may be due to the stresses in the microregion. Figure 8f is an enlargement of Figure 8d, which shows that there are mostly deep and dense micropores in the tough nests; a small amount of second-phase particles are interspersed. The presence of these second-phase particles relaxes the stress concentration in the tensile fracture, reduces the crack expansion rate and increases the strength of the laser-welded joint.

### 3.5. Impact Toughness

The heat–affected zone of the untreated HC420LA high−strength steel laser-welded joints has an average impact work of 27 J. After the 8.49 GW/cm^2^ laser impact power density treatment, the specimen joint’s heat-affected zone has an impact work of 37 J. Compared to untreated specimens, this constitutes an increase of 37%, which indicates that laser shock peening can improve the impact toughness of the HC420LA low-alloy high-strength steel laser−welded joints.

**Figure 9:**
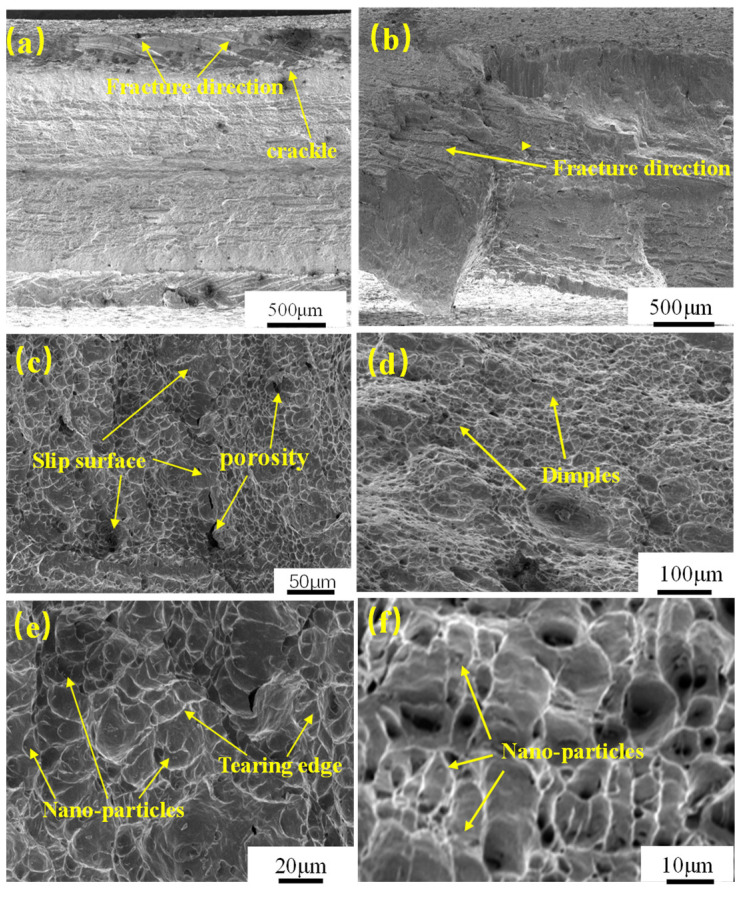
Impact toughness fracture morphology of welded joints before and after LSP. Fracture macroscopic morphology: untreated (**a**) and after LSP (**b**); fiber zone: untreated (**c**) and after LSP (**d**); fiber zone enlargement: untreated (**e**) and after LSP (**f**).

Figure 9 shows the impact fracture morphology of the heat-affected zone of the laser-welded head of HC420LA low-alloy high-strength steel before and after the laser shock peening. Figure 9a shows the impact fracture morphology of the heat-affected zone of the untreated welded joint. The fracture direction is the direction of the pendulum impact and cracks appear in the subsurface layer. As in Figure 9b which shows the fracture morphology after laser shock peening, a number of uneven “step-type” fracture slips belonging to the deconstruction and microporous aggregation-type mixed fractures, begin to surface. The slip surface is perpendicular to the pendulum impact direction: this is the slip of the material to overcome the surface potential and work function of the fracture surface, indicating the increased resistance to impact fracture. Figure 9c shows the fiber zone of the impact fracture of the untreated specimen with a large number of toughness fractures characterized by tearing ridges with microporosity of the toughness nests and a small number of swirls and slip surfaces. Combined with other scholars’ studies [24,25] that explain this phenomenon and according to the translational–rotational–vortex theory, the local occurrence of plastic deformation when the specimen is subjected to impact loading will accumulate a large number of defects in the subsurface and around the notch, thus forming a macroscopic crack due to the vortex effect of plastic flow. This forms a rotational-shear deformation zone owing to the rapid development of the local fracture. The number of tough nests shown in Figure 9d increased when compared to the number of untreated specimens, and a morphology of small tough nests surrounding large tough nests is observed. This is because the large size of the second phase particles makes them more brittle and cannot participate in the plastic deformation of the welded joint, leading to the formation of small tough nest around large tough nest morphological characteristics [26]. From Figure 9e, it can be seen that the untreated specimen has a large and shallow tough nest size, a certain directionality and a small number of second-phase particles distributed in the tough nest. After laser impact treatment, it can be seen in Figure 9f that the size of the tough nest becomes small and deep, and second-phase particles become entrapped in it. The tearing ribs also become short and dense from continuous long lines, increasing the resistance to fracture as the laser impact reduces the size of the tough nest hole, and the compressive stress generated under the impact load effectively inhibits crack generation.

## 4. Conclusions

This paper investigates the effects of laser shock peening on the evolution of the microstructure and mechanical properties of welded joints of HC420LA low-alloy high-strength steel; the main conclusions are as follows:

(1) After laser shock peening, the ferrite microstructure of the joint weld area in the high-power shock wave extrusion becomes elongated with a mesh distribution; bainite grains have a broken distribution in which the heat-affected zone and the parent material area of grain refinement increases;
(2) After laser shock peening on HC420LA, the surface and depth direction of microhardness, the welding residual tensile stress into residual compressive stress, and the residual stress value of the welded joints in each region increased by 2.24, 2.39 and 2.04 times when compared to the untreated specimens; microhardness values compared to untreated specimens increased by 7.3%, 30.3% and 19.4%. The microhardness and residual stress in the depth direction decreased due to the enhancement of the inverse bremsstrahlung effect, which affects the layer depth up to 0.6 mm;
(3) After laser shock peening on HC420LA, the tensile strength and heat-affected zone of low-alloy high-strength steel welded joints with Charpy impact absorbed more work than untreated specimens by 4.7% and 37%; given the tough fracture characteristics, the impact fracture is a slip surface—with tough fracture characteristics. Tensile fractures are of the microporous aggregation-type of ductile fracture characteristics. The welded joint toughness is increased due to residual pressure, the increase in residual stress and hardening layer formation.

## Figures and Tables

**Table 1:** Chemical composition (wt%) of the HC420LA specimen to be welded; the tensile strength is 535 MPa.

C	Si	Mn	S	P	Nb	Als	Ti
0.1	0.5	1.6	0.025	0.025	0.09	0.015	0.015

## Data Availability

The data and material used are not publicly available. We solemnly declare that this paper is the result of our research. This paper does not contain any work published or written by any other individual or group, except for the content specifically noted and cited in the paper. We fully realize that the legal consequences of this statement shall be borne by us.
